# Epidemiology and Genomic characteristics of arenavirus in rodents from the southeast coast of P.R. China

**DOI:** 10.1186/s12917-023-03798-8

**Published:** 2023-11-29

**Authors:** Qinghua Xie, Changqiang Zhu, Lele Ai, Danyue Nie, Yifan Wu, Chongcai Wang, Ji He, Weilong Tan, Lingling Zhang

**Affiliations:** 1 State Key Laboratory of Ecological Pest Control for Fujian and Taiwan Crops & Key Laboratory of Biopesticide and Chemical Biology of Ministry of Education, Fujian Agriculture and Forestry University, Fuzhou, 350002 China; 2 Nanjing Bioengineering (Gene) Technology Center for Medicines, Nanjing, 210002 China; 3 Hainan International Travel Healthcare Center, Haikou, 570311 China; 4 Xiamen International Travel Healthcare Center, (Xiamen Customs Port Outpatient Department), Xiamen, Fujian 361012 China

**Keywords:** Wenzhou virus, Rodents, Epidemiology, Genomic characterization, Southeast coastal region

## Abstract

**Background:**

Wenzhou virus (WENV), a member of the *Mammarenavirus* genus in the *Arenaviridae* family, has been detected in wild rodents from eight provinces in China, including Zhejiang, Shandong, Hainan, Xinjiang, Hunan, Guangdong, Yunnan, and Jiangxi provinces, and some countries from Southeast Asia. The IgG-antibodies of WENV have been detected in both healthy populations and patients with unknown fever and respiratory symptoms. However, the potential harmfulness of WENV to humans has been underestimated due to mild symptoms after infection, similar to respiratory diseases. Thus, it is imperative to enhance the surveillance of WENV in wild rodents, particularly *Rattus norvegicus*, and continuously monitor its prevalence.

**Results:**

From 2017 to 2021, a total of 390 wild rodents were collected from six provinces in the eastern and southern coastal areas, containing nine species of rats. Samples of each tissue were collected, and PCR amplified for identification. Four *R. norvegicus* samples were detected to be WENV-positive. No genomic sequence of WENV was detected in *Rattus flavipectus*, *Rattus losea*, *Suncus murinus*, *Apodemus agrarius*, *Mus musculus*, *Microtus fortis*, *Micromys minutus*, and *Niviventer niviventer* from Jiangsu, Zhejiang, Fujian, Hainan, Guangdong and Guangxi provinces. Three genomic sequences were identified to be WENV by phylogenetic analysis. The full-length sequences of HAIKOU-40 were amplified in *R. norvegicus* from Hainan, which showed a close relationship to Wufeng/ WFS, sharing 84.5–89.4% homology at the nucleotide level and 91.6–98.9% homology at the amino acid level. Phylogenetic analysis revealed that HAIKOU-40 formed an Asia-specific cluster with all WENVs and Loie River mammarenavirus (LORV), provisionally named Asian ancestry. This cluster has diverged earlier from the remaining mammarenavirus. The sequences obtained in Xiamen, Fujian province showed more than 90% nucleotide identities with WENV, which may be a strain of WENV. Additionally, the sequence of Wuxi-87 which was a positive sequence detected in Wuxi, Jiangsu province exhibited 83% nucleotide identity with Lassa virus (LASV). Further efforts will be made to isolate and identify this virus strain, verify the relationship between Wuxi-87 and LASV, and confirm whether *R. norvegicus* is a new host of LASV.

**Conclusions:**

In this study, we conducted a systematic examination of the prevalence of WENV among rodents on the southeast coast of China. Additionally, we characterized the genome of a newly discovered WENV strain, that confirmed the role of *R. norvegicus* in the transmission of WENV. This highlights the importance of investigating the prevalence of WENV in both wild rodents and humans.

**Supplementary Information:**

The online version contains supplementary material available at 10.1186/s12917-023-03798-8.

## Background

Three species of mammarenavirus have been reported in Asia: lymphocytic choriomeningitis mammarenavirus (LCMV), Wenzhou virus (WENV) and Loie River mammarenavirus (LORV) [1]. WENV is the most recently discovered member in the *Mammarenavirus* genus of the *Arenaviridae* family. WENV was first identified in 2015 in Wenzhou, Zhejiang province [1]. It was also been detected in wild rodents from Yunnan, Guangdong, and Hainan provinces in China [2–4]. The WENV genome consists primarily of two fragments: a 7.2-kb-long L fragment that encodes RNA-dependent RNA polymerases (RdRp) and zinc binding matrix protein (ZP), and a 3.5-kb-long S fragment that encodes nucleoprotein (NP) and glycoprotein precursor (GPC) [5]. The RdRp gene, which is part of the genome, is highly conserved among mammarenaviruses but it can vary among strains and be utilized to distinguish different strains [3].

Disease caused by mammarenavirus is predominantly prevalent in Africa and Latin America [6]. Competitive ELISA detected WENV NP-specific IgG-antibodies in 830 healthy individuals aged 0–70 years, with an overall positive rate of 4.6% (38/830) [7]. The highest positive rate was 5.4% (17/317) in people aged 45–59 years, followed by 4.1% (4/98) in people aged 60 years and over. The third age group (15–44 years old) had a positive rate of 3.6% (8/221). WENV antibodies were even detected in the sera of children under 15 years of age, with a positive rate of 1.5% [7]. From 2019 to 2020, the seroprevalence of WENV in patients with unexplained fevers in Yunnan province of China was 2.8% (23/828) as determined by competitive enzyme-linked immunosorbent assay [7, 8]. Some members of the mammarenavirus can cause severe hemorrhagic fever, such as LASV, Junín virus (JUNV), Guanarito virus (GTOV), Machupo virus (MACV), and Sabía virus (SABV) [9]. For instance, LASV alone causes approximately 100,000 to 300,000 infections with about 5,000 deaths per year (Lassa Fever CDC, 2022, https://www.cdc.gov/vhf/lassa/index.html). Therefore, newly discovered mammarenavirus like WENV, may also pose a significant threat to public safety [6]. Consequently, it is crucial to investigate the transmission routes of WENV through experimental models to prevent the emergence of these viruses.

Wild rodents are natural hosts for a variety of pathogens, such as hantavirus and paramyxovirus [6]. Previous studies have detected WENV primarily in *R. norvegicus*, *Mus musculus*, *Rattus flavipectus*, *Rattus exulans*, *Niviventer niviventer*, *Suncus murinus* [2, 10], indicating a wide range of potential hosts of WENV. demonstrated Mammarenavirus can be transmitted to humans through excrements like feces and urine, as well as bodily fluids such as saliva and blood [11–13]. The virus can spread through mucosal surfaces, including the skin, gastrointestinal tract, and respiratory tract, when humans come into contact with the feces or secretions of these animals [6]. However, the symptoms and risks associated with WENV infection in humans are still unclear, and there is a lack of corresponding control measures. Therefore, it is crucial to enhance the detection and diagnosis of viruses in both wild rodents and humans.

The high population density, extensive trade, abundant rodent species, and substantial overlap between human and rodent habitats on the southeast coast increase the risk of human exposure to WENV. In 2015, WENV was first identified in rodents and shrews in Wenzhou, Zhejiang province, China. Positive cases were observed in 15.4% of *R. flavipectus*, 11.8% of *Rattus losea*, 17.1% of *R. norvegicus*, 75.0% of *Rattus rattus*, 1.9% of *N. niviventer* and 4.4% of *S. murinus* [1, 5]. The virus has also been found in *R. norvegicus* from Xinjiang, Shandong, and Hainan provinces [4, 6, 14]. In the previous study, WENV was detected in *R. losea* in Hunan Province [5, 15]. In Guangdong province, WENV was found in *R. norvegicus* and *S. murinus* with infection rates of 6.7% and 0.5%, respectively [3]. In Yunnan province, WENV was found in *R. norvegicus, Rattus tanezumi,* and *Tupaia belangeri* with infection rates of 8.3%,17.4%, 1.5%, and 10%, respectively [2, 5]. These findings suggest that WENV can be carried by a variety of wild rodents, has multiple hosts, and has the potential for cross-species transmission. However, most studies have concentrated on sampling sites within a single province, and there has been no systematic investigation of WENV carriage in rodents in the southeast coastal areas. Therefore, it is crucial to detect WENV carriage in wild rodents along the southeast coast to monitor the potential risks of human-animal infection.

## Results

### Rodents composition and distribution

**Fig. 1:**
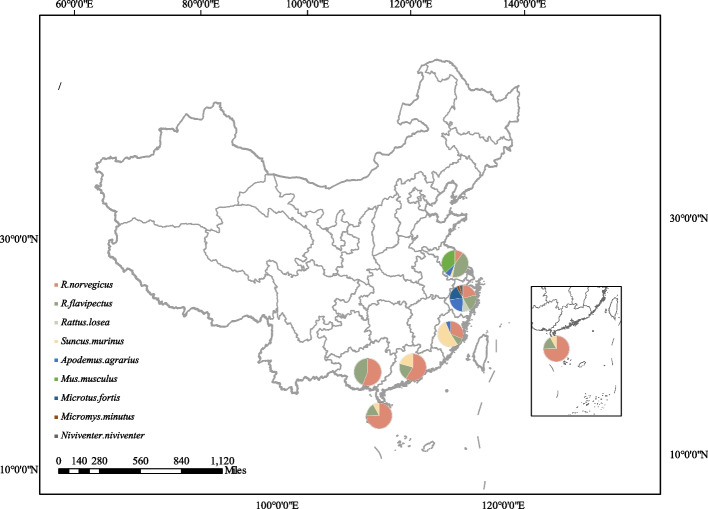
Detection of WENV in rodent-like animal samples from the eastern and southern coastal areas. This map was plotted by Adobe illustrator CS6 (Adobe Systems, USA)

A total of 390 wild rodents were collected from six eastern and southern coastal provinces during the period of 2017–2021 (Fig. 1). The collected rodents included *R. norvegicus*, *R. flavipectus*, *R. losea*, *S. murinus*, *Apodemus agrarius*, *M. musculus*, *Microtus fortis*, *Micromys minutus*, and *N. niviventer*. Among these species, *R. norvegicus* was the most dominant with 142 individuals, followed by *R. flavipectus* (91) and *S. murinus* (57) (Table 1).

**Table 1:** WENV detection results of rodents in the eastern and southern coastal provinces, China

Provinces	Jiangsu	Zhejiang	Fujian	Guangdong	Hainan	Guangxi	Total (P/D^a^)
Species							
*Rattus norvegicus* (P/D^a^)	1/7	0/23	2/25	0/17	1/56	0/14	4/142
*Rattus flavipectus* (P/D^a^)	0/30	0/23	0/8	0/6	0/13	0/11	0/91
*Rattus losea* (P/D^a^)	-	0/10	-	-	-	-	0/10
*Suncus murinus* (P/D^a^)	0/2	-	0/43	0/6	0/6	-	0/57
*Apodemus agrarius* (P/D^a^)	0/5	0/25	0/5	-	-	-	0/35
*Mus musculus* (P/D^a^)	0/26	-	-	-	-	-	0/26
*Microtus fortis* (P/D^a^)	-	0/20	-	-	-	-	0/20
*Micromys minutus* (P/D^a^)	-	0/4	-	-	-	-	0/4
*Niviventer niviventer* (P/D^a^)	-	0/5	-	-	-	-	0/5
Total (P/D^a^)	1/70	0/110	2/81	0/29	1/75	0/25	4/390

*D* detected sample numbers

^*a*^*P* positive numbers

In terms nucleic acid testing, four positive samples (1.03%, 4/390) were detected. These included one positive sample from Wuxi City, Jiangsu province (1.43%, 1/70), two positive samples from Xiamen City, Fujian Province (2.5%, 2/81) and one positive sample from Haikou City, Hainan province (1.3%, 1/75). All positive samples were obtained from *R. norvegicus*. The WENV positive nucleic acid sequences Xiamen-10 and Xiamen-13 were derived from intestinal tissues, while the WENV positive nucleic acid sequences, Wuxi-87 and HAIKOU-40 were derived from spleen tissues.

### Characterization of L genes of the three WENV strains

**Fig. 2:**
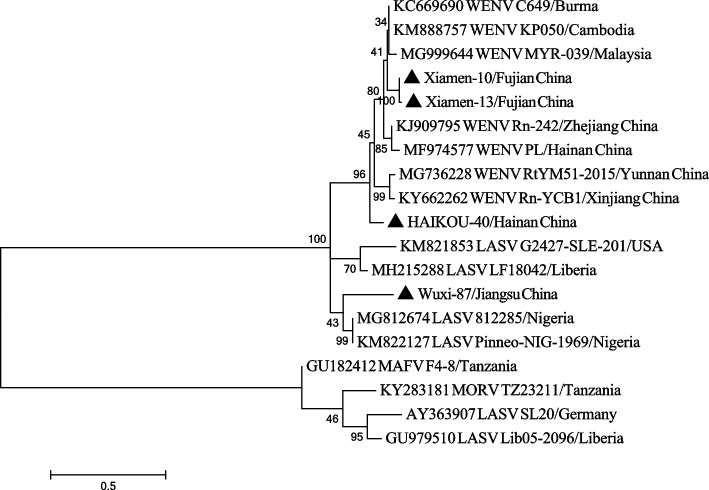
Species identification based on phylogenetic analysis of PCR amplifying products (partial RdRp gene). The phylogenetic tree was constructed using ML methods based on GTR model with gamma distribution and invariant sites, and tested with 1000 bootstrap replicates. Sequences were identified by the GenBank accession number and the strain name, followed by their origin. The sequences of the four positive samples were indicated with black triangles

Using DNASTAR software, we analyzed the sequence homology of the conserved RdRp gene in four WENV strains. The nucleotide homology of the four positive samples ranged from 60.9 to 94.7% (Table S1). Among these samples, Xiamen-10, Xiamen-13 and HAIKOU-40 showed a homology of 82.7% to 100% with other WENV sequences (Table S2). The positive sample Wuxi-87 exhibited a homology of 60.9% to 65.4% with other WENV strains (Table S2). In the phylogenetic tree analysis of the partial RdRp gene, Xiamen-10 and Xiamen-13 were found to be in the same branch, and clustered together with WENV strains isolated from Myanmar and Cambodia (Fig. 2). HAIKOU-40 formed an independent branch of evolution, while being grouped with other WENV strains; Wuxi-87 gathered with LASV and was independent of other WENV strains (Fig. 2).

### Whole-genome sequence analysis of the HAIKOU-40 strain

After NGS, the contig sequences of HAIKOU-40 were compared and spliced to obtain the full length of the S and L fragments, which were 3318 nt and 7139 nt, respectively. These sequences were uploaded to the GenBank database with the accession numbers OP562167 and OP562168 respectively.

Each complete fragment contains two open reading frames (ORFs). The coding regions were identified using the NCBI online open reading frame (ORF) finder (https://www.ncbi.nlm.nih.gov/orffinder/). The L segment codes for a 276 nt (92 aa) ZP and a 6,687 nt (2,229 aa) RdRp, linked by a 75 nt intergenic region (IGR). The S segment encodes a 1,704 nt (567 aa) NP and a 1,479 nt (492 aa) GPC, linked by a 63 nt IGR. It is worth noting that the IGR of the L segment in HAIKOU-40 is only 75 nt, which is much shorter than other WENV strains (approximately 119 nt) [16].

To characterize the relationship between HAIKOU-40 and several reported arenavirus genomes, we compared the full-length L and S fragments, RdRp gene, Z gene, NP gene and GPC gene of HAIKOU-40 with these virus strains, one by one. We analyzed the nucleotide and amino acid sequence similarities (Table 2), and found that HAIKOU-40 sequences were most closely related to the Wufeng/ WFS (Genbank accession number MZ328246.1), with 84.5–89.4% nt and 91.6–98.9% aa identity. We also observed a high sequence homology between HAIKOU-40 and the Cambodian strain C617 obtained from influenza-like patients, with the nucleotide sequence homologies of 84.6% and 87.6% for the L and S fragments, respectively.

**Table 2:** Comparison of nucleotide and amino acid similarity between HAIKOU-40 and other Mammarenavirus (%)

place		L	RdRp		Z		S	NP		GPC	
strains		nt	nt	aa	nt	aa	nt	nt	aa	nt	aa
WENV	Wufeng/ WFS	84.5	86.7	91.6	91.3	98.9	89.4	38.2	97.4	89.5	95.3
	Rn242/ Zhejiang	84.0	84.1	90.8	80.1	85.7	87.7	38.8	95.1	88.8	94.6
	Rn366/ Zhejiang	84.6	84.6	90.8	89.1	92.3	87.0	37.4	9.8	37.4	10.7
	RnGz40-2018/ Guangdong	84.5	84.3	90.0	90.9	96.7	86.2	38.9	94.9	85.6	92.7
	CH50/ Guangdong	84.2	84.4	90.0	91.3	97.8	-	-	-	-	-
	CH31/ Guangdong	-	-	-	-	-	85.7	38.7	94.4	86.5	93.8
	RnYCB1/ Xinjiang	84.0	83.9	90.3	86.6	94.5	86.0	39.3	94.2	86.0	94.0
	9–24/ Yunnan	84.3	84.2	89.1	90.6	97.8	87.5	38.7	95.9	86.8	94.0
	RtYM16-2015/ Yunnan	84.6	84.6	90.3	85.9	91.2	86.4	39.4	95.1	85.7	93.4
	Haikou/ Hainan	84.7	84.7	90.8	87.7	92.3	87.0	39.9	95.4	87.5	94.6
	PL/Hainan	84.7	84.6	90.6	87.7	92.3	-	-	-	-	-
	DK/Hainan	-	-	-	-	-	86.5	39.8	94.4	86.8	93.1
	G107/ Shandong	84.4	84.6	90.9	88.8	89.0	86.8	39.3	93.7	86.3	93.6
	MYR-039/ Malaysia	85.0	84.9	90.8	87.3	91.2	86.6	39.4	94.2	86.0	93.6
	C649 /Burma	84.6	84.5	90.7	89.9	96.7	87.5	39.0	95.4	86.3	93.6
	C617/ Burma	84.6	84.5	90.8	89.5	95.6	87.6	39.2	95.2	86.8	94.0
LORV	R4397/ Tailand	64.6	64.1	66.9	68.8	74.7	72.0	36.9	83.4	69.9	79.9
LASV	Nig08A41/ Nigeria	57.5	56.8	53.4	60.4	64.0	66.4	37.0	73.0	66.3	73.1
LCMV	53b/Spain	55.0	53.7	47.6	58.1	52.2	61.2	37.5	65.2	58.7	55.1
JUNV	XJ44/ Argentine	48.7	49.1	36.2	50.9	38.6	54.2	36.5	52.2	52.0	43.7

^a^The reference sequences accession number and sampling sites were indicated in Table S3

### Phylogenetic analysis of WENV sequences

**Fig. 3:**
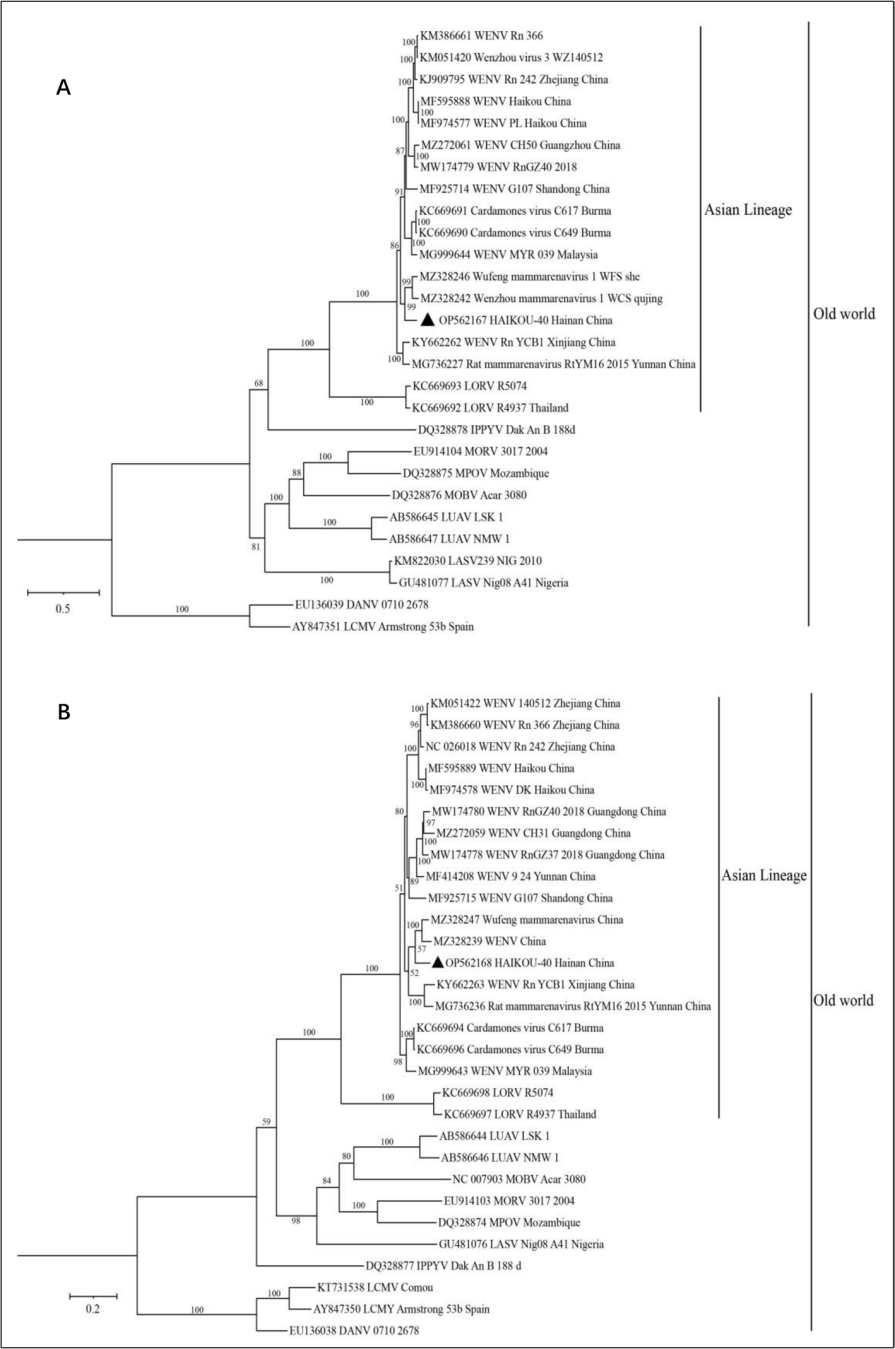
The maximum likelihood trees based on the full-length genomic sequences of WENV. The phylogenetic trees of the L (**A**) and S (**B**) segments of WENV were constructed with IQ-TREE, using automated model selection, and bootstrapped using 1000 ultrafast bootstraps. Sequences were identified by the GenBank accession number and the strain name, followed by their origin. The newly identified WENV strain in Haikou was indicated with black triangles. The reference sequences are from Genbank and their GenBank accession numbers and the strain names are shown in Table S3

After comparing the sequences of representatives of the Old World group in mammarenavirus with the sequences of HAIKOU-40, we constructed maximum likelihood trees for the S and L fragments. Figure 3 shows that the topological structures of the two phylogenetic trees are similar. The newly discovered HAIKOU-40 strain formed an Asia-specific cluster along with WENV strains from China and LORV, which we have tentatively named Asian ancestry (Fig. 3). HAIKOU-40 clustered with other WENV strains but formed a separate small clade.

## Discussion

Since its discovery in Wenzhou, Zhejiang province in 2014, WENV has been detected in wild rodents in eight Chinese provinces, primarily in *R. norvegicus*. Among the available studies, *R. norvegicus* tested positive for WENV at rates of 17.4% in Yunnan province and 17.1% in Wenzhou, Zhejiang province [1, 2]. WENV has also been found in *R. norvegicus* in Shandong, Hainan, Xinjiang, and Guangdong provinces [4, 6, 14]. These studies indicate that *R. norvegicus* has higher carriage rates of WENV compared to other rodent species [5]. This present study examined 390 wild rodents from six southeastern coastal provinces, aiming to detect the presence of WENV in wild rodents along the southeast coast. In the present study, we indentified four WENV-positive samples in *R. norvegicus*. Two samples were obtained from intestinal tissues and the other two from spleen tissues. Our finding align with Tan et al.’s research, which also detected WENV exclusively in the spleen tissue of *R. norvegicus*.

Lassa fever, which was discovered in 1969, has primarily been endemic in West African countries such as Guinea, Liberia, Sierra Leone, and Nigeria until 2020 [17, 18]. Currently, there have been no reported cases of Lassa fever in China. However, with the increasing frequency of exchanges between China and Africa, there is a higher possibility of African Lassa fever being imported into China. It is important to note that, there are no reports of LASV-infected animals being imported from endemic areas into China [17, 19, 20]. The primary source and host of LASV in nature are rodents, particularily *Mastomys natolensis*, followed by *Hylomyscus pamfi* and *Mastomys erythroleucu* [17, 21]. There have been no reports on the detection and isolation of LASV strains from *R. norvegicus*. In this study, we detected a suspected LASV nucleotide fragment (Wuxi-87) in *R. norvegicus* in Wuxi City, Jiangsu province, Wui-87 exhibited the highest nucleotide sequence homology with LASV, suggesting that it may be a member of the LASV. In the next phase, we will further examine the genetic evolution of the Wuxi-87 genome sequence and isolate the virus in the laboratory to obtain complete sequences. Simultaneously, we will monitor the prevalence of mammarenavirus in rodents on a larger scale. This research will further insights into the relationship between Wuxi-87 and LASV, and determine whether *R. norvegicus* is a new host of LASV. The identification of suspected LASV sequences in China emphasizes the importance of strengthening animal quarantine procedures for incoming ships and aircraft in regions with significant human exchanges between China and Africa, particularly in cities located at entry ports. It is also crucial to improve Lassa virus detection capabilities. Additionally, this study has discovered the presence of mammarenavirus in *R. norvegicus* for the first time in Wuxi, Jiangsu province, and Xiamen, Fujian province. These findings suggest that *R. norvegicus* serves as the primary natural host of WENV.

*Rattus norvegicus*, a member of the rodentia family, is a relatively large rodent that can be found in both domestic and wild environments. It has the ability to spread across the world through various mode of transportation [22]. In farmers' grain storage, *R. norvegicus* is a common and harmful creature [23, 24]. Changes in climate and food sources can lead to rodent migration, including crossbreeding between house mice and wild rodents, which in turn can accelerate the spread of rodent-borne diseases. Certain mammarenaviruses, such as LASV, GTOV, and JUNV have the potencial for zoonotic transmission [6, 9]. Studies have shown that LASV can be transmitted to humans not only through the feces of infected rodents, but also though human-to-human transmission [25, 26]. WENV strains have been detected and isolated from various wild rodents in China, with *R. norvegicus* having the highest detection rate. WENV has been detected in *R. norvegicus* in six provinces in China [1, 2]. In order to analyze the relationship between the HAIKOU-40 strain and representative WENV strains, we compared the nucleotide sequences of their S and L fragments. The HAIKOU-40 strain exhibited 84% nucleotide homology with the WENV reference strains found in all provinces of China (Table 2), but less than 75% homology when compared to other mammarenaviruses. According to the virus classification committee, the HAIKOU-40 strain is classified as belonging to the WENV species. The homology comparison in Table 2 revealed that HAIKOU-40 exhibits more than 15% variation in the L fragment and more than 10% variation in the S fragment, indicating a better preservation of the S fragment in WENV. In the phylogenetic analysis of the L and S fragments, the HAIKOU-40 strain falls within the same branch as the Wufeng/WFS strain (Fig. 3). The nucleotide and amino acid sequences of these two virus strains show the highest homology, suggesting a close relationship between HAIKOU-40 and Wufeng/WFS. However, the geographic origin of the Wufeng/WFS strain remains unknown.

WENV stands out from other Arenavirus due to its ability to be transmitted by multiple rodent species that share habitats with humans. This greatly increases the chancesof WENV transmission within the population. In addition, the competitive ELISA method detected WENV’s antibodies in 636 blood samples from Beijing and Shandong provinces, resulting in a positivity rate of 4.6%. This rate was compared to a rate of 2.8% among 828 unexplained fever patients in Yunnan province [7, 27]. These finding suggest that WENV has become endemic in the population. WENV has been found to cause diffuse pneumonia in rats and small cranial cavities in *R. exulans*, similar to the microcephaly observed in LCMV. This suggests a potential teratogenic effect of WENV [6, 16, 28, 29]. However, the symptoms of WENV after human infection and its potential harmfulness have been underestimated since its discovery. This underestimation is due to the predominantly mild symptoms resembling respiratory diseases. Therefore, there is an urgent need to enhance our understanding of the pathogenicity of WENV and its mechanisms, improve surveillance for WENV in wild rodents, particularly *R. norvegicus*, and determine the prevalence of WENV within the general population.

## Conclusion

This study systematically investigated the prevalence of WENV in rodents in the southeastern coast area of China. Furthermore, we conducted a genome characterization of a newly discovered WENV strain, and identified *R. norvegicus* as a significant carrier of WENV in this region. These findings emphasize the need to enhance WENV monitoring in wild rodent populations.

## Methods

### Sample Processing

To capture rodent-like animals, mouse traps were baited with doughnuts and peanuts. Sticky boards were placed every 15–20 m, in the evening and retrieved in the morning. Euthanasia of the rodents was performed by administering isoflurane through inhalation. The collected animals were dissected in a Class 2 negative pressure biosafety cabinet. Tissue samples including heart, liver, spleen, lung, kidney, intestine, brain, and muscle were taken from the animals and placed into labeled Eppendorf (EP) tubes., Subsequently, the animal samples were stored at -80 °C.

Tissue samples of rodents were collected from coastal cities and port areas of Guangxi province, Hainan province, Guangdong province, Fujian province, Zhejiang province and Jiangsu province between 2017 and 2021. Each tissue sample, approximately 1 g in size, was cut and placed into a 2 ml grinding tube. The tube contained 4 specially designed steel balls of different diameters, and 100 μL of 1xPBS (Beyotime, C0221A) was added. The tube was then sealed. After centrifugation, the supernatant was transferred to a new 1.5 mL EP tube, and supplemented with 600 μL of 1xPBS solution. The collected liquid was used for nucleic acid extraction and species identification. Total viral RNA was extracted using the MiniBEST Viral RNA/DNA Extraction Kit (Takara, Code No.9766) and RNA from each tissue sample was transcribed into cDNA for WENV detection following the instructions of the PrimeScript II 1st Strand cDNA Synthesis Kit (Takara, Code No.6210A).

### PCR detection of WENV nucleic acids

The synthetic primers LVL3359plus-WZ: AGAATYAGTGAAAGGGARAGCAATTC and LVL3754minus-WZ: CACATCATTGGTCCCCATTTACTRTGATC were used to amplify conserved regions in the RdRp gene sequence, targeting a particle size of 367 bp. The cDNA was amplified using the Ex Taq enzyme, with the following PCR parameters: an initial step of 3 min at 94℃, followed by 40 cycles of 30 s at 94℃, 30 s at 55℃, 30 s at 72℃ and a final extension step of 10 min at 72℃. The amplified sample was validated through 1.3% agarose gel electrophoresis and the positive PCR product was sent to Shenggong Biological Engineering Co., LTD.

### Whole genome sequencing of viruses

The supernatant of spleen tissues and intestinal tissues that tested positive for WENV were sent to the Beijing Institute of Genomics, Chinese Academy of Sciences for whole-genome sequencing. Initially, 140 μL of supernatant from the intestinal tissues (HAIKOU-40) was reserved for RNA extraction using the MiniBEST Viral RNA/DNA Extraction Kit. A probe-captured technique was employed to remove rodents' nucleic acid. The remaining RNA was then reverse-transcribed into cDNA, followed by second-strand synthesis. Subsequently, a library of DNA was constructed from the synthetic double-stranded DNA through DNA fragmentation, end-repair, adaptor-ligation, and PCR amplification. The Invitrogen Qubit 2.0 Fluorometer (ThermoFisher, Foster City, CA, USA) was used to quantify the library, and the quantified double-stranded DNA library was converted to a single-stranded circular DNA library by DNA denaturation and circularization. To ensure the sequencing reaction had the required signal intensity, the original single-stranded circular DNA was used as a template, and the Rolling Circle Amplification (RCA) reaction was carried out using RCA polymerase. The product of amplification was DNA nanoball (DNB) [30]. The quantification of DNBs was performed using Qubit 2.0 and they were loaded onto the flow cell, followed by sequencing using PE150 on the MGI-2000 platform (MGI, Shenzhen, China).

### Genomic and phylogenetic analysis

Reference sequences of mammarenavirus genus viruses were obtained from GenBank and listed in Table S3. Multiple sequence comparisons were conducted using the MEGA X software package [31]. Homology analysis between DNA sequences was performed using Megalign in DNASTAR software. Phylogenetic analysis was carried out using the maximum likelihood (ML) method. The phylogenetic tree of the four positive specimens was constructed based on the general time reversible model with gamma distribution and invariant sites. The tree was tested with 1000 bootstrap replicates using the MEGA X program. The genetic distance was determined using the *p*-distance with transitions and transversions method [5, 32]. The phylogenetic trees of the L and S segments of WENV were constructed using IQ-TREE, with automated model selection. The trees were then bootstrapped using 1000 ultrafast bootstraps [33]. To amplify mouse 16S ribosomal RNA gene, genomic DNA extracted from mouse muscle tissue was used as a template. The target fragment length was approximately 220 bp, with the forward primer being HedF: AYAAGACGAGAAGACCC and the reverse primer being HedR: GATTGCGCTGTTATTCC. cDNA was amplified using Ex Taq enzyme, and the PCR parameters were as follows: an initial step at 95°C for 4 minutes, followed by 35 cycles of 95°C for 40 seconds, 53 °C for 40 seconds, and 72 °C for 40 seconds. Finally, a final extension step was performed at 72 °C for 10 minutes. The samples were verified by 1.3% agarose gel electrophoresis, and the positive PCR products were sent to Shenggong Biological for sequencing. The obtained sequences were compared online using BLAST to determine the species of the sample.

### Supplementary Information

**Additional file 1: Supplementary Table 1.** Sequences distances of four WENV positive samples.

**Additional file 2: Supplementary Table 2.** Sequences distances between four WENV positive samples and other WENV strains.

**Additional file 3: Supplementary Table 3.** The reference sequences used in this study.

**Additional file 4: Supplementary Table 4.** The partial L gene sequences (367 bp) from WENV positive samples.

## Data Availability

All data generated or analyzed in this study are included in the manuscript and the supplementary information files. The complete genome sequences obtained in this study have been submitted to GenBank, accession numbers: OP562167 (https://www.ncbi.nlm.nih.gov/nuccore/OP562167) and OP562168 (https://www.ncbi.nlm.nih.gov/nuccore/OP562168). the accession numbers of partial L gene sequences (367 bp) from WENV positive samples are Xiamen-10 (OP723868, https://www.ncbi.nlm.nih.gov/nuccore/OP723868), Xiamen-13 (OP723869, https://www.ncbi.nlm.nih.gov/nuccore/OP723869), Wuxi-87 (OP723870, https://www.ncbi.nlm.nih.gov/nuccore/OP723870), and HAIKOU-40 (OP723871, https://www.ncbi.nlm.nih.gov/nuccore/OP723871).

## Abbreviations

WENV: Wenzhou virus
OW: Old World arenaviruses
GPC: Glycoprotein precursor
NP: Nucleoprotein
RdRp: RNA-dependent RNA polymerase
ZP: Zinc binding matrix protein
ORF: Open reading frames
aa: Amino acid
nt: Nucleotide
IGR: Noncoding intergenic region
LASV: Lassa virus
LORV: Loei River virus
LCMV: Lymphocytic choriomeningitis virus
GTOV: Guanarito virus
JUNV: Junín virus
ML: Maximum likelihood method
*R.**norvegicus*: *Rattus* *norvegicus*
*M.**musculus*: *Mus* *musculus*
*R.flavipectus*: *Rattus* *flavipectus*
*R.**exulans*: *Rattus* *exulans*
*N.**niviventer*: *Niviventer* *niviventer*
*S.**murinus*: *Suncus* *murinus*
*R.losea*: *Rattus* *losea*
*R.**rattus*: *Rattus* *rattus*
EP: Eppendorf
