# Supplementary material for: Epidemiology and Genomic characteristics of arenavirus in rodents from the southeast coast of P.R. China
Source: BMC Vet Res. 2023 Nov 29;19:253. doi: 10.1186/s12917-023-03798-8 (PMC10685642; doi:10.1186/s12917-023-03798-8)
Supplement: Supplementary file 1 — Additional file 1: Supplementary Table 1. Sequences distances of four WENV positive samples. [file 12917_2023_3798_MOESM1_ESM.docx]

Supplementary Table 1. Sequences distances of four WENV positive samples

|  | Percent Identity | | | | | |  |
| --- | --- | --- | --- | --- | --- | --- | --- |
|  |  | **1** | **2** | **3** | **4** |  |  |
| Divergence | **1** | *** | 94.7 | 63.6 | 88.1 | **1** | XIAMEN-10(OP723868) |
|  | **2** | 5.5 | *** | 62.3 | 83.8 | **2** | XIAMEN-13(OP723869) |
|  | **3** | 50.4 | 53.2 | *** | 60.9 | **3** | WUXI-87(OP723870) |
|  | **4** | 13.5 | 18.6 | 56.8 | *** | **4** | HAIKOU-40(OP723871) |
|  |  | **1** | **2** | **3** | **4** |  |  |
