# Supplementary material for: Epidemiology and Genomic characteristics of arenavirus in rodents from the southeast coast of P.R. China
Source: BMC Vet Res. 2023 Nov 29;19:253. doi: 10.1186/s12917-023-03798-8 (PMC10685642; doi:10.1186/s12917-023-03798-8)
Supplement: Supplementary file 2 — Additional file 2: Supplementary Table 2. Sequences distances between four WENV positive samples and other WENV strains. [file 12917_2023_3798_MOESM2_ESM.docx]

Supplementary Table 2. Sequences distances between four WENV positive samples and other WENV strains

|  |  | Percent Identity | | | | | | | | | | | | | | | | | | | |  |  |
| --- | --- | --- | --- | --- | --- | --- | --- | --- | --- | --- | --- | --- | --- | --- | --- | --- | --- | --- | --- | --- | --- | --- | --- |
|  |  | 1 | 2 | 3 | 4 | 5 | 6 | 7 | 8 | 9 | 10 | 11 | 12 | 13 | 14 | 15 | 16 | 17 | 18 | 19 | 20 |  |  |
| Divergence | 1 | *** | 94.7 | 63.6 | 88.1 | 95.1 | 94.8 | 93.5 | 77.1 | 77.1 | 88.1 | 91.9 | 90.4 | 89.8 | 89.8 | 89.5 | 88.9 | 88.9 | 86.6 | 90.8 | 89.8 | 1 | XIAMEN-10(OP723868) |
|  | 2 | 5.5 | *** | 62.3 | 83.8 | 90.5 | 89.9 | 88.9 | 76.3 | 76.3 | 83.8 | 89.5 | 88.0 | 87.4 | 84.9 | 84.7 | 84.4 | 84.1 | 82.7 | 87.0 | 85.2 | 2 | XIAMEN-13(OP723869) |
|  | 3 | 50.4 | 53.2 | *** | 60.9 | 87.0 | 87.3 | 87.5 | 77.9 | 77.9 | 100.0 | 85.9 | 87.1 | 87.7 | 85.2 | 87.8 | 87.8 | 88.1 | 91.2 | 87.6 | 85.2 | 3 | HAIKOU-40(OP723871) |
|  | 4 | 13.5 | 18.6 | 56.8 |  | 63.1 | 63.1 | 63.4 | 82.4 | 82.4 | 60.9 | 65.4 | 64.5 | 65.1 | 62.9 | 62.9 | 63.1 | 64.3 | 62.3 | 62.8 | 62.9 | 4 | WUXI-87(OP723870) |
|  | 5 | 5.1 | 10.2 | 51.6 | 15.0 |  | 99.1 | 93.4 | 76.3 | 76.3 | 87.0 | 92.1 | 91.5 | 90.9 | 90.8 | 89.6 | 90.8 | 88.8 | 87.0 | 91.8 | 91.6 | 5 | WENV/C617(KC669691) |
|  | 6 | 5.5 | 10.9 | 51.6 | 14.6 | 0.9 |  | 93.1 | 77.1 | 77.1 | 87.3 | 91.5 | 91.5 | 90.9 | 89.9 | 89.3 | 90.5 | 89.0 | 86.2 | 91.8 | 90.8 | 6 | WENV/C649(KC669690) |
|  | 7 | 7.0 | 12.1 | 51.0 | 14.3 | 7.1 | 7.4 |  | 75.6 | 75.6 | 87.5 | 91.3 | 91.3 | 90.7 | 90.1 | 88.9 | 90.3 | 89.2 | 87.5 | 91.0 | 91.5 | 7 | WENV/MYR-039(MG999644) |
|  | 8 | 28.2 | 29.3 | 20.1 | 27.1 | 29.6 | 28.4 | 30.9 |  | 100.0 | 77.9 | 76.3 | 77.9 | 78.6 | 77.1 | 77.1 | 78.6 | 77.1 | 79.4 | 78.6 | 77.1 | 8 | LASV/812285(MG812674) |
|  | 9 | 28.2 | 29.3 | 20.1 | 27.1 | 29.6 | 28.4 | 30.9 | 0.0 |  | 77.9 | 76.3 | 77.9 | 78.6 | 77.1 | 77.1 | 78.6 | 77.1 | 79.4 | 78.6 | 77.1 | 9 | LASV/Pinneo-NIG-1969(KM822127) |
|  | 10 | 13.5 | 18.6 | 56.8 | 0.0 | 15.0 | 14.6 | 14.3 | 27.1 | 27.1 |  | 85.9 | 87.1 | 87.7 | 85.2 | 87.8 | 87.8 | 88.1 | 91.2 | 87.6 | 85.2 | 10 | WENV/Haikou(MF595888) |
|  | 11 | 8.8 | 11.5 | 47.3 | 16.5 | 8.6 | 9.3 | 9.5 | 29.6 | 29.6 | 16.5 |  | 93.1 | 92.5 | 92.8 | 88.3 | 91.9 | 87.4 | 86.8 | 92.8 | 92.8 | 11 | WENV/G107(MF925714) |
|  | 12 | 10.6 | 13.3 | 49.4 | 14.8 | 9.3 | 9.3 | 9.5 | 27.3 | 27.3 | 14.8 | 7.4 |  | 98.2 | 93.4 | 88.6 | 95.8 | 88.3 | 87.4 | 99.7 | 93.4 | 12 | WENV/Rn-242(KJ909795) |
|  | 13 | 11.3 | 14.1 | 48.0 | 14.1 | 10.0 | 10.0 | 10.3 | 26.1 | 26.1 | 14.1 | 8.1 | 1.8 |  | 92.8 | 89.5 | 95.2 | 89.8 | 87.4 | 97.9 | 92.8 | 13 | WENV/Rn-366(KM386661) |
|  | 14 | 11.3 | 17.1 | 52.1 | 17.3 | 10.1 | 11.2 | 11.0 | 28.5 | 28.5 | 17.3 | 7.7 | 7.0 | 7.8 |  | 87.8 | 92.9 | 87.2 | 87.8 | 92.8 | 93.5 | 14 | WENV/CH50(MZ272061) |
|  | 15 | 11.7 | 17.5 | 52.2 | 13.8 | 11.5 | 11.9 | 12.4 | 28.0 | 28.0 | 13.8 | 13.2 | 12.8 | 11.7 | 13.8 |  | 88.6 | 96.3 | 88.9 | 88.7 | 88.6 | 15 | WENV/RtYM16-2015(MG736227) |
|  | 16 | 12.4 | 17.8 | 51.6 | 13.8 | 10.1 | 10.5 | 10.7 | 26.2 | 26.2 | 13.8 | 8.8 | 4.4 | 5.0 | 7.6 | 12.7 |  | 88.1 | 87.0 | 95.4 | 92.6 | 16 | WENV/Haikou(MF974577) |
|  | 17 | 12.4 | 18.2 | 49.1 | 13.4 | 12.5 | 12.2 | 12.0 | 27.7 | 27.7 | 13.4 | 14.3 | 13.1 | 11.2 | 14.5 | 3.8 | 13.4 |  | 88.4 | 88.7 | 88.1 | 17 | WENV/Rn-YCB1(KY662262) |
|  | 18 | 15.4 | 20.2 | 9.6 | 53.7 | 14.9 | 16.1 | 14.3 | 24.6 | 24.6 | 9.6 | 15.2 | 14.3 | 14.4 | 13.8 | 12.4 | 14.9 | 13.1 |  | 87.3 | 86.6 | 18 | WENV/Wufeng(MZ328246) |
|  | 19 | 10.1 | 14.4 | 14.2 | 52.5 | 8.9 | 8.9 | 9.8 | 26.1 | 26.1 | 14.2 | 7.7 | 0.3 | 2.1 | 7.8 | 12.6 | 4.8 | 12.5 | 14.5 |  | 93.1 | 19 | WENV/WZ140510(MF925714) |
|  | 20 | 11.3 | 16.7 | 17.3 | 52.1 | 9.1 | 10.1 | 9.3 | 28.5 | 26.5 | 17.3 | 7.7 | 7.0 | 7.8 | 7.0 | 12.7 | 7.9 | 13.4 | 15.3 | 7.4 |  | 20 | WENV/9-24(MF414207) |
|  |  | 1 | 2 | 3 | 4 | 5 | 6 | 7 | 8 | 9 | 10 | 11 | 12 | 13 | 14 | 15 | 16 | 17 | 18 | 19 | 20 |  |  |
