# Supplementary material for: Epidemiology and Genomic characteristics of arenavirus in rodents from the southeast coast of P.R. China
Source: BMC Vet Res. 2023 Nov 29;19:253. doi: 10.1186/s12917-023-03798-8 (PMC10685642; doi:10.1186/s12917-023-03798-8)
Supplement: Supplementary file 3 — Additional file 3: Supplementary Table 3. The reference sequences used in this study. [file 12917_2023_3798_MOESM3_ESM.pdf]

Supplementary Table 3. The reference sequences used in this study

	Strains	GenBank accession no. L	GenBank Accession no. S	Complete genome or partial gene	Sampling sites
WENV	Wufeng WFS	MZ328246	MZ328247	Complete genome	China
	WCS	MZ328242	MZ328239	Complete genome	China
	Rn 242	KJ909795	NC_026018	Complete genome	Zhejiang, China
	140512	KM051420	KM051422	Complete genome	Zhejiang, China
	Rn 366	KM386661	KM386660	Complete genome	Zhejiang, China
	RnGZ40 2018	MW174779	MW174780	Complete genome	Guangdong, China
	CH50	MZ272061	-	Complete genome	Guangdong, China
	Rn YCB1	KY662262	KY662263	Complete genome	Xinjiang, China
	RtYM51-2015	-	MG736228	Part genome	Yunnan, China
	9-24	-	MF414208	Complete genome	Yunnan, China
	RtYM16-2015	MG736227	MG736236	Complete genome	Yunnan, China
	Haikou	MF595888	MF595889	Complete genome	Hainan, China
	PL	MF974577	-	Complete genome	Hainan, China
	G107	MF925714	MF925715	Complete genome	Shandong, China
	KP050	KM888575	-	Part genome	Cambodia
	MYR 039	MG999644	MG999643	Complete genome	Malaysia
	C649	KC669690	KC669696	Complete genome	Burma
	C617	KC669691	KC669694	Complete genome	Burma
LORV	R4937	KC669692	KC669697	Complete genome	Tailand
	R5074	KC669693	KC669698	Complete genome	Thailand
LASV	Nig08 A41	GU481077	GU481076	Complete genome	Nigeria
	NIG 2010	KM822030	-	Complete genome	Nigeria
	G2427-SLE-201	KM8218531	-	Part genome	USA
	LF18042	MH215288	-	Part genome	Liberia
	812285	MG812674	-	Part genome	Nigeria
	Pinneo-NIG-1969	KM822127	-	Part genome	Nigeria
	SL20	AY363907	-	Part genome	Germany
	Lib05-2096	GU979510	-	Part genome	Liberia
LCMV	53b	AY847351	AY847350	Complete genome	Spain
	Comou	-	KT731538	Complete genome	French
DANV	0710 2678	EU136039	EU136038	Complete genome	USA
LUAV	LSK 1	AB586645	AB586644	Complete genome	Zambia
	NMW 1	AB586647	AB586646	Complete genome	Zambia
IPPYV	Dak An B 188d	DQ328878	DQ328877	Complete genome	France
MORV	3017 2004	EU914104	EU914103	Complete genome	Tanzania
	TZ23211	KY283181	-	Part genome	Tanzania
MPOV	Mozambique	DQ328875	DQ328874	Complete genome	Mozambique
MOBV	Acar 3080	DQ328876	NC_007903	Complete genome	France
MAFV	F4-8	GU182412	-	Part genome	Tanzania
