# Supplementary material for: Epidemiology and Genomic characteristics of arenavirus in rodents from the southeast coast of P.R. China
Source: BMC Vet Res. 2023 Nov 29;19:253. doi: 10.1186/s12917-023-03798-8 (PMC10685642; doi:10.1186/s12917-023-03798-8)
Supplement: Supplementary file 4 — Additional file 4: Supplementary Table 4. The partial L gene sequences (367 bp) from WENV positive samples. [file 12917_2023_3798_MOESM4_ESM.pdf]

Supplementary Table 4. the partial L gene sequences (367 bp) from WENV positive samples.

Strains	Sampling sites	Species	Sequences
XIAMEN-10	Fujian Province	*Rattus norvegicus*	TTTGTGGGCAAGAGACACAGAGAGCCTCATGGCTATCAGGGCATTCTCAAACTCCTTTTCATCATTTAAACAACTCCCTCTGAAGTGTTTGGTGAATGCCTCAAAGTAATCTTCTATCAACCTTGTGTACATTTTGGTTCTGAGGTCACCAATGTATAACTCCCGATTCCCCCCAACCTGCTCTTTATAAGACAAAGAAAATTTCAACCTTCCAGTGTTTGGGCCAACAGATGTGAAAGACTCTGGTGACTCTTCACTATAAAAGCACAGGTTCTTTAAGGCCGCACTAGTGCAGTTTGAGAGGCTCAATGCCTTACTTAGTGCTTCTGAATTGCTCTCCCTTTCACTGATTCTAA
XIAMEN-13	Fujian Province	*Rattus norvegicus*	CTTGGGGGTCTCAGACACCCATTAGCCTCATGGCTATCCTGGCATTCTCAAACTCCTTTTCATCATTTATACAACTCCCTCTGAAGTGTTTGGTGAATGCCTCATAGTAATCTTCTATCAACCTTGTGTACATTTTGGTTCTGAGGTCACCAATGTATAACTCCCGATTCCCCCCAACCTGCTCTTTATAAGACAAAGAAAATTTCAACCTTCCAGTGTTTGGGCCAACAGATGTGAAAGACTCTGGTGACTCTTCACTATAAAAGCACAGGTTCTTTAAGGCCGCACTATTGCATTTTGAGAGGCTCAATGCCTTACTTAGTGCTTCCGAATTGCTCTCCCTTTCCCTCATTCTAT
WUXI-87	Jiangsu Province	*Rattus norvegicus*	CTCAAGGGGGGCTCTTCCTCCGTGCTACTCTTCCATTCATTGCCCCTTATTATTTTCAATGCTGATTCATCCTCCCAACATGAAGTTTTAAATGACTTGGTTAACCTCTCAAAATAATCTTCTAAGAGCCTAGTGAACATTTTGGTTCTAAGATCTCCAATATAAAGTTCTCTATTACCTCCCAATTGTTCTTTATAAGAGAGTCCAAATTTTAATCTTCCTGTATCTGGTCCAACTGAAGTATAAGACTTAGGTGATTCACATGAATAAAAGCATAAGTTTTTTAAGGCAGCACTAACACAATCCGTTAGATTTAGGGCTTCTGATATAGCTTGTGAATTGCTCTCCCTTTCACTGATTCT

Strains	Sampling sites	Species	Sequences
HAIKOU-40	Hainan Province	*Rattus norvegicus*	TGGGCAAGAGAAACAGAAAGCCTCATAGCTATCAGGGCATTCTCAAACTCTTTCTCATCATTTAAACAGCTCCCTCTAAAATGTTTAGTAAATGCCTCGAAGTAATCCTCTATCAACCTCGTATACATCTTGGTCCTGAGATCACCGATGTATAGCTCTCGATTTCCTCCAACTTGCTCTTTGTAAGACAATGAGAATTTCAACCTCCCGGTGTTTGGGCCAACAGATGTGAAAGACTCTGGTGACTCCTCACTGTAAAAACACAGATTCTTCAAGGCTGCACTAGTGCAATTTGAGAGGCTCAATGCCTTACTCAGTGCCTCTGAATTACTTTCCCTCTCACTAATTCTGACATCATCTAG
